# Supplementary material for: Genetic Signatures for Distinguishing Chemo-Sensitive from Chemo-Resistant Responders in Prostate Cancer Patients
Source: Curr Issues Mol Biol. 2024 Mar 11;46(3):2263–77. doi: 10.3390/cimb46030145 (PMC10969469; doi:10.3390/cimb46030145)
Supplement: Supplementary file 1 [file cimb-46-00145-s001.zip › cimb-2889497-supplementary.pdf]

Table S1

| ITEM TO CHECK | IMPORTANCE | Comments |
|---|---|---|
| **EXPERIMENTAL DESIGN** | | |
| Definition of experimental and control groups | E | Table 1 |
| Number within each group | E | 4 Good responders & 5 Poor responders. Each patient with both normal and tumour tissue. |
| Assay carried out by core lab or investigator's lab? | D | Core lab |
| **SAMPLE** | | |
| Description | E | FFPE tissues |
| Volume/mass of sample processed | D | 10 to 30μm |
| Microdissection or macrodissection | E | microdissection |
| Processing procedure | E | Materials and Methods |
| Sample storage conditions and duration (especially for FFPE samples) | E | Stored at Room Temperature |
| **NUCLEIC ACID EXTRACTION** | | |
| Procedure and/or instrumentation | E | Materials and Methods |
| Name of kit and details of any modifications | E | Quick-DNA/RNA™ FFPE Kit (Zymo Research) |
| Details of DNase or RNAse treatment | E | on-column DNase I treatment (Quick-DNA/RNA™ FFPE Kit) |
| Contamination assessment (DNA or RNA) | E | DNA contamination assessed by qPCR without RT step. |
| Nucleic acid quantification | E | Qubit RNA High Sensitivity Kit |
| Instrument and method | E | Qubit 4 Fluorometer |
| Purity (A260/A280) | D | Assessed by NanoDrop 8000 spectrophotometer |
| RNA integrity method/instrument | E | Agilent High Sensitivity ScreenTape and 4200 TapeStation Syst |
| RIN/RQI or Cq of 3' and 5' transcripts | E | RIN values of RNA samples and Cq values of 3' and 5' transcriptsdetailed in Table S1 |
| Inhibition testing (Cq dilutions, spike or other) | E | Inhibition assessed by SPUD qPCR assay, Table S2 |
| **REVERSE TRANSCRIPTION** | | |
| Complete reaction conditions | E | cDNA was synthesized from FFPE total RNA using the Maxima H Minus cDNA Synthesis MasterMix with dsDNase Kit (Thermo Fisher) according to the manufacturer's protocol. All samples were prepared in 200 μL PCR-strip tubes. Approximately 100 ng of RNA was mixed with 1 μL of 10x dsDNase Buffer and 1 μL of dsDNase and diluted up to 10 μL with Nuclease-free water. Each sample was mixed gently and centrifuged briefly. All samples were then incubated in the Applied Biosystems 2720 Thermal Cycler (Thermo Fisher) at 37 °C for 2 min and stored on ice. Each dsDNase-treated RNA sample was then mixed with 4 μL of 5x Maxima cDNA H Minus Synthesis MasterMix and 6 μL of Nuclease-free water and incubated in a Applied Biosystems 2720 Thermal Cycler (Thermo Fisher) at the following settings: 10 min at 25 °C for priming, 15 min at 50 °C for reverse transcription to occur, 5 min at 85 °C for inactivation of reverse transcriptase. No-RT control samples were created at the same time in which RNA from the each FFPE sample was mixed with Maxima No RT Control mastermix containing no Maxima H Minus Reverse Transcriptase. A No template control (NTC) reaction was also included in which reverse trancription was carried out without any RNA template. |
| Amount of RNA and reaction volume | E | 100 ng of RNA in a 20 μL of volume per sample. |
| Priming oligonucleotide (if using GSP) and concentration | E | No GSP was used. The Maxima H Minus cDNA Synthesis Master Mix used in this study contains oligo(dT) and random hexamers for priming cDNA synthesis. |
| Reverse transcriptase and concentration | E | Maxima H Minus cDNA Synthesis MasterMix with dsDNase Kit (Thermo Fisher) |
| Temperature and time | E | Specified in 'Complete reaction conditions' |
| Manufacturer of reagents and catalogue numbers | D | |
| Cqs with and without RT | D* | In all experiments, no Cq values were obtained for samples without RT compared to samples with RT. |
| Storage conditions of cDNA | D | All cDNA samples were stored at -20 °C. |
| **qPCR TARGET INFORMATION** | | |
| If multiplex, efficiency and LOD of each assay. | E | Not Applicable |
| Sequence accession number | E | **ABCB1:** NM_001348945.2; **CYP1B:** NM_000104.4 |
| Location of amplicon | D | |
| Amplicon length | E | Table 2 |
| *In silico* specificity screen (BLAST, etc) | E | Primer specificity confirmed b Primer-BLAST |
| Pseudogenes, retropseudogenes or other homologs? | D | |
| Sequence alignment | D | |
| Secondary structure analysis of amplicon | D | |
| Location of each primer by exon or intron (if applicable) | E | Not Applicable |
| What splice variants are targeted? | E | No splice variants were targeted. |
| **qPCR OLIGONUCLEOTIDES** | | |
| Primer sequences | E | Table 2 |
| Probe sequences | D** | Not Applicable |
| Location and identity of any modifications | E | None |
| Manufacturer of oligonucleotides | D | Integrated DNA Technologies |
| Purification method | D | Standard Desalting |

| | | |
|---|---|---|
| **qPCR PROTOCOL** | | |
| Complete reaction conditions | E | qPCR was performed in a total reaction volume of 10 µL consisting of 2 µl diluted preamplified cDNA as template, 5µL of 2x PowerUp SYBR Green I Mastermix (Thermo Fisher), and both the forward and reverse primers to a final concentration of 500 nM. NTC reactions were included in all assays as negative controls. Each reaction was run in triplicate. Reactions were performed on the QuantStudio 12K Flex Real-Time PCR System (Applied Biosystems) using the following cycling parameters: 50°C for 2 min; initial denaturation at 95°C for 2 min followed by 40 cycles of 95°C for 15 secs and 57°C for 1 min. A melt curve analysis was performed on all reactions at the end of the PCR run using default parameters. Amplification data were analyzed with Life Technologies QuantStudio 12K Flex Software v1.2.4, applying user-defined thresholds to obtain Cq-values. Outliers in technical replicate reactions, reactions that showed no amplification and reactions that showed multiple Tm peaks during melt curve analysis were removed from analysis. Data was finally exported into Excel spreadsheets for further analysis. |
| Reaction volume and amount of cDNA/DNA | E | 10 µL reaction with 2 µL diluted preamplified cDNA |
| Primer, (probe), Mg++ and dNTP concentrations | E | 500 nM each primer with PowerUp SYBR Green Master Mix (Thermo Fisher) |
| Polymerase identity and concentration | E | PowerUp SYBR Green Master Mix (Thermo Fisher) |
| Buffer/kit identity and manufacturer | E | PowerUp SYBR Green Master Mix (Thermo Fisher) |
| Exact chemical constitution of the buffer | D | PowerUp SYBR Green Master Mix (Thermo Fisher) |
| Additives (SYBR Green I, DMSO, etc.) | E | PowerUp SYBR Green Master Mix (Thermo Fisher) |
| Manufacturer of plates/tubes and catalog number | D | MicroAmp™ Optical 384-Well Reaction Plate with Barcode, Catalog number: 4326270 |
| Complete thermocycling parameters | E | See 'Complete reaction conditions' |
| Reaction setup (manual/robotic) | D | Manual |
| Manufacturer of qPCR instrument | E | Applied Biosystems QuantStudio™ 12K Flex thermocycler |
| **qPCR VALIDATION** | | |
| Specificity (gel, sequence, melt, or digest) | E | Confirmed by melt curve analysis (single peak) for each primer set and gel electrophoresis |
| For SYBR Green I, Cq of the NTC | E | Cq of NTC was 'undetermined' |
| Standard curves with slope and y-intercept | E | Available on request |
| PCR efficiency calculated from slope | E | ABCB1, E= 98.4%; CYP1B: E= 108.1%; GAPDH, E= 99.7%; HPRT, E=92.9%; HSPCB, E=103.9% |
| r2 of standard curve | E | > 0,98 for all assays |
| **DATA ANALYSIS** | | |
| qPCR analysis program (source, version) | E | QuantStudio™ 12K Flex Software 1.5 |
| Cq method determination | E | Automatic baseline setting and manual threshold setting |
| Outlier identification and disposition | E | A Cq value was discarded if the melt curve was inconsistent with other samples in the experiment. |
| Results of NTCs | E | No Cq values were assigned to NTC for all assays except for HSPCB where Cq for NTC was > 10 cycles away from the experimental samples |
| Justification of number and choice of reference genes | E | The stability of three reference genes: GAPDH, HPRT and HSPCB was evaluated using RefFinder. Both GAPDH and HSPCB were used to normalized the qPCR data. |
| Description of normalisation method | E | qPCR data were analyzed in Microsoft Excel using both GAPDH and HSPCB as normalization controls and the Pfaffl (Pfaffl, 2001) method to calculate relative expression. |
| Number and stage (RT or qPCR) of technical replicates | E | 3 technical qPCR replicates |
| Repeatability (intra-assay variation) | E | Standard deviation of triplicates < 0.5 |
| Statistical methods for result significance | E | A significance level of p<0.05 was used |
| Software (source, version) | E | Microsoft Excel, SAS Version 9.2 |

**Table.S2. Primer nucleotide sequences and product lengths for the target and reference genes amplification by qPCR**

| Gene Symbol | Primer Name | Primer Sequence (5'------ 3') | Amplicon Size | Reference |
|---|---|---|---|---|
| ABCB1 | 3-abcb1Fp1<br>3-abcb1Rp1 | GGA CTG TAA CTG ACT GCC TTG C<br>GGC AGT TTG GAC AAG ATG ACT CC | 124 bp | This study |
| CYP1B1 | CYP1B1pp3F<br>CYP1B1pp3R | GCTGCAGTGGCTGCTCCT<br>CCCACGACCTGATCCAATTCT | 81 bp | (Finnström et al. 2001) |
| GAPDH | 3-GAPDHFp<br>3-GAPDHRp | AGT CCC TGC CAC ACT CAG<br>TAC TTT ATT GAT GGT ACA TGA CAA GG | 123 bp | (Nolan et al. 2006) |
| HPRT1 | HPRT1F<br>HPRT1R | GAC CAG TCA ACA GGG GAC AT<br>CCT GAC CAA GGA AAG CAA AG | 132 bp | (Liu et al. 2015) |
| HSPCB | HSPCBF<br>HSPCBR | TCT GGG TAT CGG AAA GCA AGC C<br>GTG CAC TTC CTC AGG CAT CTT G | 80 bp | (Jacob et al. 2013) |

**Table. S3:** Molecular function and network analysis following gene enrichment.

| Molecular function and pathways | Library | p-value | q-value | z-score | Combined score |
|---|---|---|---|---|---|
| MicroRNAs in cancer | KEGG_2021_Human | 0.0002395 | 0.002155 | 39380 | 328300 |
| Nuclear Receptors Meta-Pathway WP2882 | WikiPathway_2021_Human | 0.0002536 | 0.004599 | 39362 | 325900 |
| Sulindac Metabolic Pathway WP2542 | WikiPathway_2021_Human | 0.0004999 | 0.004599 | 4999 | 37990 |
| PMC5346035 Nihms844046F1 | PFOCR_Pathways | 0.0004999 | 0.002168 | 4999 | 37990 |
| PMC4844852 Nihms-744712-F0001 | PFOCR_Pathways | 0.0004999 | 0.002168 | 4999 | 37990 |
| PMC7235470 RMV-30-e2109-g001 | PFOCR_Pathways | 0.0004999 | 0.002168 | 4999 | 37990 |
| PMC5953846 Nihms963973F8 | PFOCR_Pathways | 0.0004999 | 0.002168 | 4999 | 37990 |
| PMC5896366 Dmd.118.080663F2 | PFOCR_Pathways | 0.0004999 | 0.002168 | 4999 | 37990 |
| LncRNA-mediated mechanisms of therapeutic resistance WP3672 | WikiPathway_2021_Human | 0.0005999 | 0.004599 | 3999 | 29660 |
| Benzo(a)pyrene metabolism WP696 | WikiPathway_2021_Human | 0.0008998 | 0.004827 | 2499 | 17520 |
| Estrogen Receptor Pathway WP2881 | WikiPathway_2021_Human | 0.0013 | 0.004827 | 1666 | 11070 |
| Tryptophan metabolism | KEGG_2021_Human | 0.004196 | 0.01096 | 486.8 | 2664 |
| ABC transporters | KEGG_2021_Human | 0.004495 | 0.01096 | 453.5 | 2451 |
| Ovarian steroidogenesis | KEGG_2021_Human | 0.005094 | 0.01096 | 399 | 2106 |
| Steroid hormone biosynthesis | KEGG_2021_Human | 0.006091 | 0.01096 | 332.3 | 1695 |
